# Increased Listening Effort: Is Hearing Training a Solution?—Results of a Pilot Study on Individualized Computer-Based Auditory Training in Subjects Not (Yet) Fitted with Hearing Aids

**DOI:** 10.3390/audiolres15050124

**Published:** 2025-09-27

**Authors:** Dominik Péus, Jan-Patric Schmid, Andreas Koj, Andreas Radeloff, Michael Schulte

**Affiliations:** 1 Universitätsklinik für Hals-Nasen-Ohrenheilkunde, Charité Universitätsmedizin Berlin, 12203 Berlin, Germany; 2 Universitätsklinik für Hals-Nasen-Ohrenheilkunde, Carl von Ossietzky Universität, 26122 Oldenburg, Germany; 3 KOJ Hearing Network, 77855 Achern, Germany; 4 Hörzentrum Oldenburg GmbH, 26122 Oldenburg, Germany

**Keywords:** cognitive auditory training, auditory training, cognition, hearing loss, listening effort, computer-based cognitive auditory training, CCAT

## Abstract

**Background:** Hearing and cognition decline with age. Hearing is now considered an independent risk factor for later cognitive impairment. Computerized cognitive auditory training is being discussed as a possible adjunctive therapy approach. **Objectives**: The aim of this exploratory study is to investigate how the success of a computer-based cognitive auditory training (CCAT) can be measured. For this purpose, the influence of a CCAT on different dimensions of hearing and cognition was determined. **Materials and Methods**: 23 subjects between 52 and 77 years old were recruited with normacusis to moderate hearing loss. They underwent 40 digital training lessons at home. Before, during, and after completion, concentration ability with the d2-R, memory (VLMT), subjective hearing impairment (HHI), hearing quality (SSQ12), listening effort in noise (ACALES), and speech understanding in noise (GÖSA) were measured. **Results and Discussion**: In this uncontrolled, non-randomized study, one of the main findings was that cognitive dimensions, namely processing speed, improved by 12.11 ± 16.40 points (*p* = 0.006), and concentration performance improved by 12.56 ± 13.50 points (*p* = 0.001), which were not directly trained in CCAT. Learning performance also improved slightly by 4.00 ± 7.00 (*p* = 0.019). Subjective hearing handicap significantly reduced by 10.70 ± 12.38 (*p* = 0.001). There were no significant changes in the SSQ-12 (*p* = 0.979). Hearing effort improved by 1.79 ± 2.13 dB SPL (*p* = 0.001), 1.75 ± 2.09 (*p* = 0.001), and 3.32 ± 3.27 dB (*p* < 0.001), respectively. Speech understanding in noise did not improve significantly. CCAT is likely to improve several dimensions of hearing and cognition. Controlled future studies are needed to investigate its efficacy.

## 1. Introduction

The prevalence of hearing loss increases with age: around 20% of 60- to 69-year-olds and 40% of 70- to 79-year-olds in Germany are affected by hearing loss [1]. Although hearing aids can help people with hearing loss to hear speech, their ability to listen and understand semantically may still be suboptimal. Cognitive domains such as perception, thinking, knowledge, or memory deteriorate significantly with age [2].

However, the causal mechanism explaining this association between hearing loss and cognitive decline remains controversial. Panza et al. [3] and Pabst et al. [4] were able to show that hearing loss is a significant risk factor for cognitive impairment or can at least contribute to it manifesting earlier or to a greater extent. In particular, hearing loss is associated with an increased incidence of dementia in older adults, which in turn leads to poorer processing of sensory information [3,4,5]. In older adults, sensory loss often accompanies cognitive impairment, reducing the ability to process information needed for daily activities [6].

However, early detection and intervention for existing hearing loss could potentially have a positive impact on cognitive performance and reduce the risk of dementia and cognitive impairment in old age [7,8]. Possible interventions include hearing rehabilitation with hearing aids [7] or, in specific cases, hearing implants such as cochlear implants [9].

Many hearing-impaired people report increased difficulty understanding speech. However, hearing tests commonly used in everyday clinical practice, such as tone audiograms, speech tests such as the Freiburg monosyllable speech test in quiet or in noise in simple standard configurations, often capture only a limited, peripheral aspect of these primarily subjective complaints at best. Understanding speech is an active process that extends beyond passive hearing.

Especially in noise, speech intelligibility requires mental load. This component, known as ‘listening effort’, is not or only partially taken into account by speech test applications. However, it is increasingly recognized as a crucial factor, distinct from speech intelligibility [10]. McGarrigle defined listening effort as “the mental effort required to hear and understand an auditory message” [11]. Increased listening effort can have significant consequences, such as stress-related absenteeism from work [12]. Reliable, well-established procedures for testing speech intelligibility exist. One method for recording listening effort is the relatively new ACALES (Adaptive CAtegorical Listening Effort Scaling) method. The ACALES method assesses listening effort adaptively, using anchors ranging from effortless to extremely strenuous by individually adjusting the signal-to-noise ratio (SNR). This allows for the determination of individual SNR ranges for the categories between effortless and extremely strenuous.

New methods such as computer-based cognitive auditory training (CCAT) [13] have already been developed to slow and prevent hearing-related cognitive decline in older adults and to reduce subjectively perceived hearing difficulties that cannot be fully addressed with hearing aids. However, cognitive auditory training should not be regarded as an alternative to hearing rehabilitation with hearing aids (hearing aids, implants), but rather as a supplement. The aims of CCAT are to improve speech comprehension, cognition, and listening skills. Research on CCAT is highly heterogeneous, partly because the programs differ considerably between manufacturers, and partly because the methods used to measure training success—and thus the study endpoints—vary widely.

To date, there has been no reliable evidence of the effectiveness of the CCAT [14]. A single test cannot adequately capture the various dimensions of hearing and cognition. Accordingly, the authors considered a combination of established auditory measurement procedures, newer procedures, and questionnaires, as well as methods borrowed from cognitive psychology, to be most suitable for investigating the CCAT.

In this pilot study, patients with subjective hearing complaints were recruited regardless of the respective hearing threshold, who did not (yet) use hearing aids, and were exposed to a commercially available CCAT. Their concentration, memory, subjective hearing impairment, hearing quality, listening effort in background noise, and speech comprehension in background noise were measured before, during, and after completion.

## 2. Methods

### 2.1. Test Subjects

23 test subjects were recruited from the database of Hörzentrum Oldenburg GmbH. Of the 23 subjects, three subjects (13.04%) terminated the study prematurely. Complete datasets from 20 subjects (average age 66.85 ± 5.86 years, 14 women and 6 men) were therefore available for the analysis. These subjects completed the training in 46 ± 5.4 days (minimum 41 and maximum 60 days).

The study was conducted at the Hörzentrum Oldenburg on behalf of KOJ hearing network GmbH, Achern, Germany, and approved by the local ethics committee (No. Drs.EK/2021/031-01). An expense allowance of 120 EUR was paid for participation in the entire study. The effort for each subject was an average of 3.75 h at the test center and 30–45 min per day for the 40-day training at home. Subjects who had subjective hearing impairment, but who had not previously been fitted with hearing aids, were recruited. Exclusion criteria were abnormal cognition or an abnormal result in the dementia screening test (DemTect value < 13) [15]. All subjects had a screening result indicating no cognitive impairment (16.15 ± 2.25).

The hearing threshold was determined using a pure-tone audiogram. On average, a better ear hearing loss of M = 25.62 dB HL was measured over the frequencies of 0.5, 1, 2, and 4 kHz (min: 16.25 dB HL and max: 38.75 dB HL). According to the new WHO definition (PTA of the better ear of <20 dB), two subjects were classified as having normal hearing, 16 subjects had mild hearing loss (PTA < 35 dB), and two subjects had moderate hearing loss. 18 subjects (95%) showed a symmetrical hearing loss with a difference of ≤10 dB HL. One subject had a difference of 12.5 dB HL, and one subject had a difference of 16.15 dB HL.

### 2.2. Procedures

The tests and questionnaires described in more detail below were carried out at three different times (see Table 1): at recruitment (T1), approximately three weeks (+/− 4 days) after the start of training (T2), and after completion of training (after approx. 6 weeks; T3).

### 2.3. Hearing Training Intervention

The subject of the cognitive hearing training was the KOJ hearing training developed by KOJ hearing network GmbH (Achern, Germany, www.koj.training, accessed on 1 June 2025). 10-inch Android tablets were used to carry out the CCAT. These tablets provide a platform for displaying interactive content and delivering the therapy. In addition, free-field loudspeakers were used, which were set to a comfortable volume. CCAT is structured into individual lessons, each of which takes about 30–45 min a day. Each lesson comprises 5 to 10 exercises designed to train specific skills, such as understanding words or memorizing numbers. These exercises consist of different tasks, such as recognizing 30 different number combinations.

The audio recordings used cover three age categories (young, middle-aged, and older) and the male and female genders, ranging from simple numbers, words, and syllables to meaningless sentences and quotations. Additionally, four audiobooks were provided, and each participant could choose one at the start to listen to in sections throughout the 40-day training period. These elements were integrated into the training to keep motivation high.

During training, the CCAT continuously adapted to the user’s individual performance and changing needs. This was achieved by adaptively adjusting the SNR as well as the selection and scope of exercises within each lesson. After each exercise, the user’s feedback was collected and compared with objective performance data. Consistent correct/incorrect rates and reaction times were used for primary assessment. To compensate for initial and fatigue effects, the first 20% of all entries and the last 10% were not included in the assessment.

In addition to the adaptive learning content, the CCAT platform offers an integrated feedback and analysis module that classifies all training data according to the following six listening disciplines: auditory comprehension (discrimination), auditory filtering (noise discrimination), memory, directional hearing, attention direction, and auditory perception. The user received simplified feedback in the form of up to six stars per category, which were combined into an overall score to visualize progress in an understandable and motivating way.

### 2.4. Procedures and Testing

#### 2.4.1. Listening Effort

Listening effort was measured using the ACALES (Adaptive CAtegorical Listening Effort Scaling) scaling method [16]. The ACALES method measures the mental listening effort, or listening effort, that a person has to exert to understand speech in background noise. The individually perceived listening effort is assessed using a 14-point scale ranging from “0 = effortless” to “13 = extremely strenuous” and the additional category “background noise only”. SNR values corresponding to extreme, moderate, and mild levels of subjective listening effort were evaluated

Two modes were examined. A “simple” situation in which the speech signal was presented from the front from a 0° direction with a stationary background noise from the same direction (S0N0). This situation is referred to below as “simple”. The “complex” situation included a speech signal from the front (0°) and a cafeteria noise from 135° (S0/N135) in order to achieve a spatial separation of speech and maskers [16]. This situation is referred to as “complex”.

#### 2.4.2. Speech Comprehension

Speech comprehension was assessed before the start and after the end of the 40-day CCAT using the Göttinger Satztest im Störschall (GÖSA) [17]. The GÖSA is a speech test with everyday sentences. Here, the speech intelligibility threshold was measured in dB SNR at which 50% of the words are understood (SRT50%). The test setup with the two different ACALES maskers was retained, so that both the “GÖSA simple” and the “GÖSA complex” were tested.

### 2.5. Questionnaires

Two hearing-specific questionnaires were administered. The first was the short version of the Speech, Spatial and Qualities of Hearing Scale (SSQ-12) questionnaire [18]. The scales of the SSQ-12 range from 0 to 10 on a visual analog scale, with higher scores indicating a better assessment of hearing abilities in various situations. The second was the Hearing Handicap Inventory (HHI) questionnaire, used in the variants for pensioners (Elderly variant HHI-E) and adults (Adults, HHI-A) with the “Social/Situational” and “Emotional” subscales [19]. Overall, a total score of 0 (no handicap) to 100 (total handicap) can be achieved, with symptom severity graded from 0–16% = no handicap, 18–42% = mild to moderate handicap, and 44%+ = significant handicap [19].

### 2.6. Cognitive Tests

The verbal learning and memory test VLMT is a test of learning and memory skills [20]. For this study, the learning performance of Dg1 to Dg5 was analyzed over all five learning sessions as a repeated measure in the form of a learning curve. A cut-off score of ≥48 points is used to distinguish between normal performance and indications of learning/memory impairment. The d2-R test is used to measure concentration in attention tasks [21]. It measures the test subject’s ability to concentrate in terms of processing time (BZO) and the speed and accuracy with which similar visual stimuli can be distinguished, which is called concentration performance (KL). BZO represents the number of processed target objects regardless of correctness, whereas KL is the number of processed target objects minus the number of errors. In the d2-R, a percentile rank of <16 indicates impairment in processing speed (BZO) and concentration performance (KL).

### 2.7. Statistics

The statistical analyses were conducted using SPSS Statistics 29.0 and using GraphPad Prism version 9. The significance level was set at *p* = 0.05.

As the Kolmogorov–Smirnov test showed no statistically significant deviation from a normal distribution for any of the main variables, all results could be analyzed using parametric tests.

To evaluate whether the variables of interest changed over time as a result of the CCAT, a two-factor repeated measures analysis of variance (RM-ANOVA) was conducted for each thematic group: (1) cognitive tests, (2) questionnaires, and (3) listening effort and speech comprehension. If the assumption of sphericity was violated, the Greenhouse–Geisser correction was used. The significance level of the repeated measures analysis of variance of α = 0.05 was Bonferroni-adjusted to α = 0.017 (α = 0.05/3). Estimated marginal means were calculated, and pairwise comparisons between time points for each variable of interest were performed with Bonferroni correction to control for multiple comparisons in each thematic group. Effect sizes were reported as partial eta squared (*_p_*η^2^).

Cognitive tests

The within-subject factors included Time (2 levels: T1 and T3) and cognitive test results (3 levels: VLMT, d2-R processing time (BZO), and d2-R concentration performance (KL)).

Questionnaires

The within-subject factors included time (2 levels: T1 and T3) and the questionnaires (2 levels: SSQ-12 and HHI).

Listening effort and speech comprehension

The within-subject factors included time (2 levels: T1 and T3) as well as listening effort and speech comprehension (8 levels: three levels of ACALES simple, three levels of ACALES complex, GÖSA simple, and GÖSA complex).

Furthermore, Cohen’s d was used to calculate the effect size. The calculation was carried out for paired samples using the difference values (Diff). Cohen’s d was then calculated as follows: d = mean (Diff)/standard deviation (Diff). A value from 0.5 to 0.8 stands for a medium effect. Values above 0.8 represent a strong effect.

## 3. Results

Table 2 presents the descriptive statistics of the sample in the variables of interest for T1 and T3, along with the results of the post hoc tests of the repeated measures analysis of variance and Cohen’s d effect sizes.

### 3.1. Cognitive Tests: VLMT and d2-R

**Figure 1:**
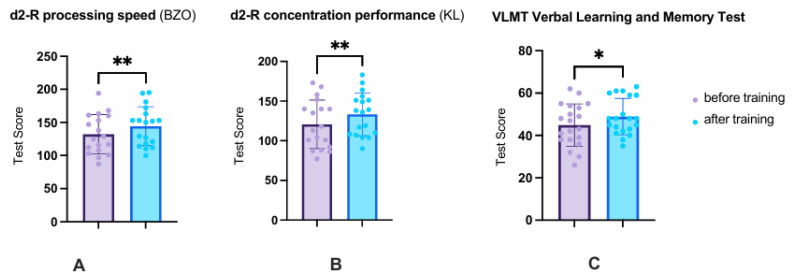
Results of the cognitive tests d2-R (processing speed (BZO) and concentration performance (KL)) and the verbal learning and memory test (VLMT). Both the main domains of the d2R and the memory ability changed positively, whereby the memory ability improved less than the BZO and the KL. The average value and standard deviation before training are shown in purple and after training in blue. Values before vs. after training * *p* < 0.05; ** *p* < 0.01.

The repeated-measures ANOVA with a Greenhouse–Geisser correction regarding cognitive tests revealed that the mean performance levels showed a statistically significant difference between measurement points (T1 vs. T3), F (1.00, 17.00) = 15.08, *p* = 0.001, *_p_*η^2^ = 0.47. The Bonferroni-adjusted post hoc analysis revealed significantly higher performance scores at T3 compared to T1 in the VLMT, d2-R processing speed (BZO), and d2-R concentration performance (KL) measures (*p* < 0.05; see Table 2 and Figure 1A,B).

The CCAT significantly improved overall list learning performance in the VLMT by 4.00 ± 7.00 (*p* = 0.019). Across all five learning sessions, Dg1 to Dg5, the test subjects were thus able to memorize an average of 4 more words after the CCAT than before the CCAT. The processing speed (BZO) improved by 12.11 ± 16.40 points (*p* = 0.006), and the most important parameter of the d2-R test, the ability to concentrate (KL), improved by 12.56 ± 13.50 (*p* = 0.001) (see Figure 1C).

At the beginning of the study (T1), 55% of subjects (*n* = 11) showed impairment in the VLMT, whereas at T3, only 45% (*n* = 9) remained impaired. Similarly, at T1, 55.6% (*n* = 10) demonstrated impaired processing speed (BZO) and 44.4% (*n* = 8) impaired concentration performance (KL). By T3, these proportions had decreased to 44.4% (*n* = 8) and 27.8% (*n* = 5), respectively.

### 3.2. Questionnaires: HHI and SSQ-12

**Figure 2:**
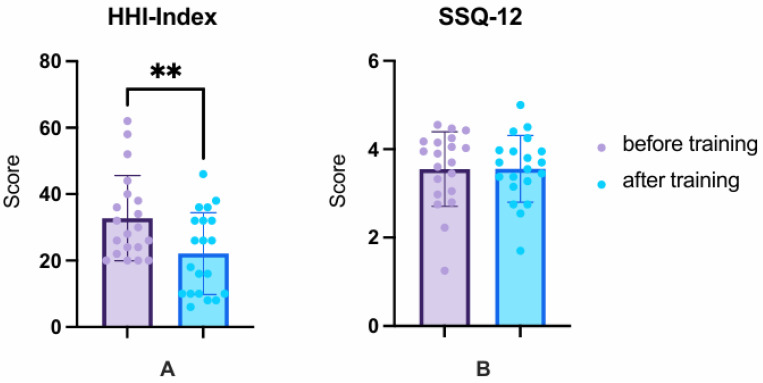
Hearing Handicap Inventory (HHI) and Spatial and Qualities of Hearing Scale (SSQ-12). The subjectively perceived hearing handicap measured with the HHI questionnaire improved significantly, whereas hearing qualities measured with the SSQ-12 did not change significantly. The average value and standard deviation before training are shown in purple and after training in blue. Values before vs. after training ** *p* < 0.01.

The repeated-measures ANOVA of the questionnaires HHI and SSQ-12 revealed a significant difference in the scores between T1 and T3, F (1.00, 19.00) = 7.47, *p* < 0.013, *_p_*η^2^ = 0.28. The Bonferroni-adjusted post hoc analysis revealed no significant change in SSQ-12 scores (*p* = 0.979; see Table 2) but showed a significant reduction in HHI scores from T1 to T3 by 10.70 ± 12.38 points (*p* = 0.001; see Table 2 and Figure 2).

**Figure 3:**
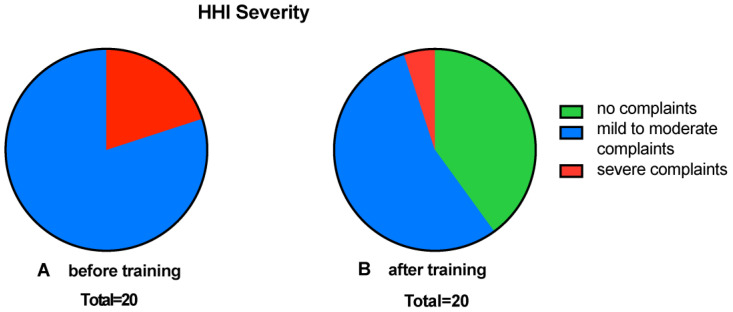
Pie charts for hearing impairment HHI. Categorically divided into no disability, mild to moderate disability, and severe disability. Shown are pie charts of the subjective degree of hearing impairment before (**A**) and after training (**B**).

Before starting the CCAT, all subjects had at least a mild to moderate handicap. In 16 (80%) subjects, there was a mild to moderate impairment, whereas 4 (20%) reported a significant impairment (Figure 3A). After the training, only 11 (55%) still stated that they had mild to moderate complaints, 8 (40%) nominally reduced their handicap to the “no handicap” category, and only 1 subject (5%) remained in the significant complaint group (Figure 3B).

### 3.3. Listening Effort and Speech Comprehension: ACALES and GÖSA

**Figure 4:**
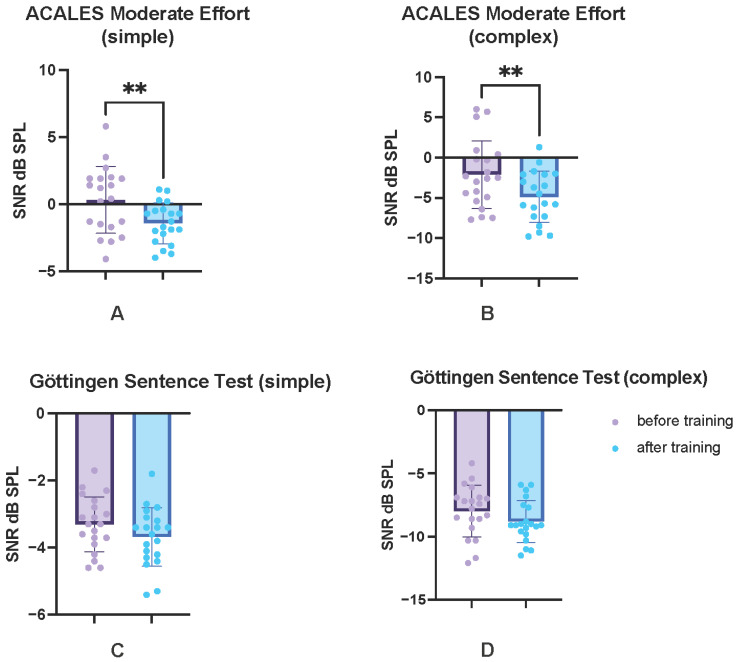
Listening effort and comprehension in noise. Results of the ACALES (Adaptive CAtegorical Listening Effort Scaling) measurement before (purple) and after training (blue), as a measure of the perceived listening effort in background noise, exemplary for the category medium effort for the two acoustic conditions “simple” (**A**) where signal and noise source was the same and “complex” (**B**), where signal and noise sources were separated. The results of the Göttinger Satztest (GÖSA) are also shown for the two acoustic conditions “simple” (**C**) and “complex” (**D**). Only the values before (T1) and after training (T3) are shown. ** *p* < 0.01.

The repeated-measures ANOVA with a Greenhouse–Geisser correction revealed a statistically significant difference between measurement time points (T1 vs. T3) for listening effort and speech comprehension, F (1.00, 19.00) = 32.23, *p* < 0.001, *_p_*η^2^ = 0.66. The Bonferroni-adjusted post hoc analysis showed descriptive, yet non-significant, changes in GÖSA speech intelligibility thresholds in noise for the S0N0 (simple) and S0N135 (complex) conditions, with SNR improvements of 0.38 ± 1.09 dB SPL (*p* = 0.137) and 0.82 ± 1.79 dB SPL (*p* = 0.054), respectively, between T1 and T3 (see Table 2 and Figure 4C,D). In contrast, post hoc tests revealed significant improvements across all ACALES variables over time (*p* < 0.05; see Table 2).

The threshold measured in dB SNR, at which it became subjectively extremely strenuous for the subject to follow the speaker in background noise, improved by 1.79 ± 2.13 dB SPL after the CCAT (*p* = 0.001). With moderate effort, the SNR improved by 1.75 ± 2.09 (*p* = 0.001; see Figure 4A), and with minimal effort by 3.32 ± 3.27 dB (*p* < 0.001). For the ACALES measured in the complex listening situation, there was a relatively constant improvement for all categories of listening effort: 2.88 ± 3.61 (*p* = 0.002), 2.75 ± 3.47 (*p* = 0.002), and 2.74 ± 3.26 (*p* = 0.001); see also Figure 4B.

### 3.4. Effect Sizes

Table 2 shows the largest effect sizes for ACALES in the simple acoustic situation, the HHI, and the concentration performance in the d2-R. All effect sizes are above 0.8 and can therefore be described as strong.

## 4. Discussion

In this method study, 40 training units of CCAT were completed over a period of 41 to 60 days. These consisted of several listening exercises, for which the test subjects needed 30–45 min.

An important aspect of investigating CCAT is considering its clinical and everyday relevance. The main problem here is that the training tasks in CCAT should not be too similar to the test task used for their evaluation. Training success can only be generalized with untrained tasks [14,22]. All of the tests used here are untrained tasks. In addition, this is one of the first studies to include tests that are independent of listening, such as the visual d2-R. It was found that possible auditory improvements after computer-based individualized hearing training were primarily in cognitive dimensions, listening effort, and hearing impairment in everyday life. In contrast, speech intelligibility and subjective hearing ability and quality (as measured with the SSQ-12) did not change significantly.

In addition to improvements in verbal learning and memory (VLMT), there were statistically significant improvements in concentration ability (KL) and processing speed (BZO) on the d2-R. These results could indicate that CCAT could not only influence the auditory dimension of hearing but could also show effects on broader cognitive functions. Similarly, Lee et al. [23] reported improvements in attention and working memory. They interpreted these effects within the framework of neuroplasticity, which posits that behavioural changes can be induced by manipulating external experiences, such as through structured training. Other researchers have proposed that cognitive–auditory training enhances the efficiency of attention allocation [24]. This is in line with Ferguson & Henshaw, who emphasize the benefits of cognitive-auditory training for executive functions (such as working memory and divided attention) [25]—which are essential for understanding speech. As most research on cognitive auditory training has focused on working memory, an executive function, evidence regarding its effects on memory is limited. Interestingly, Loewy et al. found that targeted cognitive auditory training improved verbal memory in younger individuals at clinical high risk for psychosis [26]. In a small, randomized MRI study, regional gray matter volume increases in the right dorsolateral prefrontal cortex and the left inferior temporal gyrus were observed in the auditory–cognitive training group, which was interpreted as an increased neuroplasticity compared to the control group [27]. However, it should be noted that there are also comprehensive reviews that have only found low effects for auditory training for cognitive function in adults with hearing loss [28]. Thus, due to the lack of a control group without CCAT, a learning effect through repetition of the VLMT and d2-R cannot be ruled out with certainty.

Using the ACALES adaptive test procedure, this study demonstrated a statistically verifiable improvement in perceived auditory effort after the CCAT. Depending on the level of listening effort, this improvement ranged from 1.75 to 3.27 dB (for the standard situation S0N0). In other words, the distance between the speech signal and noise after the CCAT could be reduced by this SNR to obtain the same subjective effort rating. These SNR changes were greater than the intraindividual standard deviation of the reference dataset of Krueger et al. [16] for all three anchors, effortless, moderate, and extreme. One consequence of increased listening effort can be listening-related fatigue [11]. The reduced listening effort presented here suggests that the mental effort for the test subjects improved significantly in all SNR ranges after the CCAT. This could suggest that the test subjects may have experienced less (auditory) fatigue relevant to everyday life after the CCAT. The relevance of the ACALES procedure was also demonstrated in a study by Winneke et al. [29], who showed that a new type of microphone directionality for noise reduction reduced listening effort. This was accompanied by reduced alpha-band activity (9–12 Hz) in the electroencephalogram. This indicates that brain activity changes during speech comprehension when listening effort is reduced. Lower listening effort can have important real-life implications. For instance, Kramer et al. [12] showed that the dimensions of listening effort were the greatest risk factor for stress-related absenteeism in hearing-impaired employees.

In addition to reduced listening effort, the HHI questionnaire indicated a reduction in the self-rated hearing handicap. The 20 test subjects reported a moderate to significant handicap when they were included in the study. After completing the training, 90% of subjects reported an improvement in symptoms, and eight subjects (40%) no longer had scores indicating a hearing handicap (Figure 3). The mean improvement in the HHI of 10.70 ± 12.38 points is also considerable compared to values reported in the literature. Sweetow and Sabes showed a slightly lower HHI improvement of 7.5 after training with a CCAT. However, improvements in the HHI may also reflect the subjects‘ expectations, which is why cognitive auditory training should be evaluated using different approaches.

The SSQ-12 questionnaire revealed no significant changes after the training. One possible explanation for this could be the structure of the SSQ-12. It consists of 12 questions in which very specific everyday hearing impressions are described, and the respondent is asked how he/she copes with them. These questions address different aspects of hearing, such as spatial perception, speech comprehension in noise, and sound identification. As the SSQ-12 is a shortened version of the SSQ-49, its main advantage is the shorter time required for documentation; however, this very brevity could also pose a problem, as it tests completely different qualities in just 12 questions. It remains to be clarified whether, in a larger study with possible use of the SSQ-49, in which significantly more questions relate to one hearing quality in each case, effects might not be measurable after all. In addition, the SSQ-17 as another short form of the SSQ, is also influenced by non-auditory factors such as age, gender, and education [30].

There was no significant change in the GÖSA in the present pilot study, but at best a trend towards improved speech intelligibility; this applies both to the standard version (S0N0 condition) and the test setup with spatially separated fluctuating noise (S0N90). Descriptively, the effect was slightly higher in the complex GÖSA than in the standard GÖSA. However, more complex tests may be needed, e.g., with the specification of semantic comprehension in the background noise or more complex and realistic scenarios with different background noises from different directions, in order to map the effects of a CCAT intervention. However, this will be the subject of further research. The calculated Cohen’s d effect sizes indicate that the ACALES, d2-R, and the HHI are promising tools for investigating the effects of CCATs. The high effect sizes suggest that CCATs can particularly demonstrate their benefits in these dimensions.

Adherence and compliance in the cohort of test subjects were high. Notably, 20 out of the 23 subjects (87%) completed the training in full. Sweetow and Henderson–Sabes reported a compliance rate of 73% [13], and Stacey et al. a compliance rate of 73% [31]. For adherence, it is beneficial that the individual is able to recognize tangible benefits from the training so that the hearing training is accepted and continued by the individual. Therefore, improvements in the self-reported subjective tests HHI and ACALES seem very important for the success of CCAT. Whether the high level of adherence can also be confirmed in everyday settings outside of studies remains to be seen.

### Limitations

A limitation of this pilot study is the selectivity of the sample, consisting of test subjects with predominantly mild to moderate hearing loss. None of these subjects were fitted with a hearing aid. It is currently not possible to transfer the results to people with more severe hearing loss and to people fitted with hearing aids without further ado. Additionally, the small sample size (*n* = 20) limits the generalizability and robustness of findings. Moreover, the study lacked a control group for comparison. Future research should aim for a larger sample size and include a control group to strengthen the validity of the results. Furthermore, improvements, especially in self-reported outcomes as in the HHI, may simply reflect the expectations raised by receiving an intervention. As multiple subjective measures were used, various biases (e.g., expectancy effects, social desirability) could have influenced the results [32]. Therefore, a follow-up study with a comparable dummy or placebo training, along with solely objective measures, is warranted to investigate the effectiveness of the CCAT used, which was not the focus of the present method study. However, the VLMT and the d2-R are non-trained tasks, suggesting that these cognitive variables have improved without the influence of expectations.

## 5. Conclusions

In summary, no significant effect of the application of 40 units of the CCAT on the intelligibility of speech in noise and the evaluation of listening situations in the SSQ-12 was found in the present study. Nevertheless, significant effects were observed in this sample with regard to the subjective assessment of listening effort and the HHI. With regard to the cognitive tests, significant effects were observed on verbal learning and memory skills, as well as on the ability to concentrate, with high effect sizes. These results suggest that the CCAT can positively impact well-being (in terms of hearing impairment and listening effort) and cognitive performance. However, further studies with control groups and larger sample sizes are necessary for further assessment, especially with regard to effectiveness.

## Figures and Tables

**Table 1:** Overview of the measurement procedure.

Measurement		Time 1	Time 2	Time 3
		0	3rd week	6th week
Questionnaires	HHI	x		x
	SSQ12	x		x
Cognitive Tests	d2-R	x		x
	VLMT	x		x
Audiometric Tests	Speech understanding threshold (GOESA)	x	x	x
	Subjective listening effort (ACALES)	x	x	x

x stands for measure.

**Table 2:** Study results of the tests before (time 1) and after (time 3) the training. Mean ± standard deviation. The right-hand columns show *p*-values from post hoc tests of repeated measures ANOVAs, adjusted using Bonferroni correction, as well as the effect sizes for each test (Cohen’s d).

	Time 1 (T1)	Time 3 (T3)	Post Hoc *p*-Value	Cohen’s Effect Size d
VLMT (max. 75 points)	44.90 ± 10.03	48.90 ± 8.63	0.019	0.57
d2-R processing speed (BZO; max. 258 points)	132.17 ± 29.70	144.28 ± 29.40	0.006	0.74
d2-R concentration performance (KL; max. 258 points)	120.72 ± 30.61	133.28 ± 26.98	0.001	0.93
HHI (max. 100 points)	32.80 ± 12.82	22.10 ± 12.32	0.001	0.86
SSQ12 (max. 120 points)	71.08 ± 16.88	71.15 ± 15.10	0.979	
ACALES simple [SNR dB SPL] Hearing effort (max. X points)	6.02 ± 4.02 (effortless) 0.32 ± 2.50 (moderate) −4.56 ± 2.37 (extremely strenuous)	2.70 ± 2.28 (effortless) −1.43 ± 1.51 (moderate) −6.35 ± 1.75 (extremely strenuous)	<0.001 0.001 0.001	1.01 0.84 0.84
ACALES complex [SNR dB SPL] Hearing effort (max. X points)	4.39 ± 5.22 (effortless) −2.13 ± 4.21 (moderate) −8.22 ± 4.54 (extremely strenuous)	1.52 ± 2.84 (effortless) −4.87 ± 3.19 (moderate) −10.96 ± 3.00 (extremely strenuous)	0.002 0.002 0.001	0.80 0.79 0.84
GÖSA simple [SNR dB SPL] Speech comprehension (max. dB)	−3.3 ± 0.82	−3,68 ± 0.87	0.137	
GÖSA complex [SNR dB SPL] Speech comprehension (max. dB)	−7.98 ± 2.03	−8.8 ± 1.66	0.054	

## Data Availability

The datasets analyzed during the current study are not publicly available due to data privacy limitations to but are available from the corresponding author upon reasonable request.
